# Perfluorooctanoic Acid and Its Short-Chain Substitutes Induce Cytotoxic and Prooxidative Changes in Human Peripheral Blood Mononuclear Cells: A Comparative Study

**DOI:** 10.3390/ijms26115408

**Published:** 2025-06-05

**Authors:** Izabela Kaczmarska, Katarzyna Mokra, Jaromir Michałowicz

**Affiliations:** 1 Doctoral School of Exact and Natural Sciences, University of Lodz, Matejki 21/23 St., 90-237 Lodz, Poland; izabela.kaczmarska@edu.uni.lodz.pl; 2 Department of Biophysics of Environmental Pollution, Faculty of Biology and Environmental Protection, University of Lodz, 141/143 Pomorska St., 90-236 Lodz, Poland; jaromir.michalowicz@biol.uni.lodz.pl

**Keywords:** cell viability, PBMCs, PFOA, perfluorooctanoic acid, reactive oxygen species

## Abstract

Perfluorooctanoic acid (PFOA) and its short-chain substitutes, perfluorohexanoic acid (PFHxA) and perfluorobutanoic acid (PFBA), are persistent environmental pollutants associated with widespread human exposure through occupational and environmental routes. The aim of this was to investigate the effects of PFOA, PFHxA, and PFBA on the intracellular level of adenosine-5’-triphosphate (ATP) in human peripheral blood mononuclear cells (PBMCs) and their viability, size, and granularity. Moreover, oxidative and nitrosative stress was assessed based on the levels of reactive oxygen species (ROS), reactive nitrogen species (RNS), and highly reactive oxygen species (hROS, mainly hydroxyl radical). Finally, oxidative damage to protein and lipids in PBMCs was measured. The cells were incubated for 1 h and 24 h at concentrations correlated to human occupational and environmental exposure (0.001–200 µg/mL) to the substances. Our findings indicate that PFOA and its short-chain analogs cause different effects in human PBMCs. PFOA induced statistically significant alterations almost in all studied parameters, substantially decreasing cell viability and ATP level and altering the size and granularity of tested cells; in contrast, PFHxA and PFBA induced significant changes only at some studied parameters. PFOA also induced a notable increase in intracellular ROS and RNS levels, which suggest that both oxidative stress and nitrosative stress influence its cytotoxic potential. Interestingly, the shortest-chain compound, PFBA, induced changes that were not observed for PFHxA. This suggests that the length of the chain determines the triggering of certain alterations in PBMCs. Importantly, the changes were noted at concentrations corresponding to those associated with occupational exposure. These findings contribute to our understanding of the immunotoxicity of PFOA and its substitutes, indicating the potential health risks associated with chronic exposure, particularly in populations with occupational exposure or high environmental PFOA burdens.

## 1. Introduction

Perfluoroalkyl and polyfluoroalkyl substances (PFASs) are a group of anthropogenic chemical compounds that have been widely utilized in various industries and consumer products since the late 1940s due to their unique properties, such as temperature resistance, hydrophobicity, and oleophobicity. PFASs can be found in kitchenware, food-contact materials, firefighting foams, and water- and stain-resistant fabrics, as well as in paints and flame retardants. However, the use of these products can result in the release of PFASs into the environment, leading to contamination [1].

One of the most extensively studied classes of PFASs is perfluorinated carboxylic acids (PFCAs) [2]. These compounds feature a carbon chain in which all hydrogen atoms have been replaced by fluorine atoms, with a carboxyl group at the end of the chain. The bond between carbon and fluorine is extremely strong and stable, which contributes to the thermal and chemical stability of these compounds [3]. The most frequently measured compound within the PFCA group is perfluorooctanoic acid (PFOA). PFOA exhibits toxic effects on mammalian organisms, has the ability to bioaccumulate, and is consequently resistant to degradation [2].

The bioaccumulation potential of PFOA has been attributed to the presence of the strong carbon–fluorine bonds in its structure. Its broad spectrum of toxic effects is believed to be influenced by inter alia exposure duration, exposure route, age, sex, health status, and genetic predisposition. In the liver, it induces hepatocyte apoptosis and disrupts lipid metabolism and fatty acid transport, potentially leading to hepatic steatosis and the development of neoplastic lesions such as adenomas and hepatocellular carcinoma [4]. Moreover, PFOA elevates liver enzyme activity, indicating hepatic injury [5]. In the kidneys, it exhibits nephrotoxic effects, contributing to impaired filtration function [6]. In the reproductive system, PFOA and other long-chain perfluorinated compounds reduce sperm motility and may be associated with an increased risk of more advanced stages of endometriosis, thereby decreasing fertility [7,8]. Prenatal exposure to PFOA is associated with reduced birth anthropometric measures in neonates, reflecting its teratogenic and developmental toxicity [9]. Additionally, increased blood cholesterol levels have been observed, suggesting metabolic disturbances [10].

As a result of numerous scientific studies highlighting the negative impact of PFOA on both organisms and the environment, legal regulations regarding its use have been implemented. In 2009, PFOA was included on the list of the Stockholm Convention on Persistent Organic Pollutants [11], significantly restricting its production. In the European Union, PFOA is subject to additional national regulations; for example, Norway introduced a ban on the use of PFOA in consumer products starting in 2013 [12]. The United States Environmental Protection Agency (US EPA) issued a directive to reduce PFOA production by 2010, with a complete phase-out targeted by 2015 [12]. In 2021, the US EPA classified PFOA as a “probable carcinogen” [13]. As a result, efforts have been made to identify alternative compounds with similar properties. Focus shifted to short-chain compounds from the same group of PFCAs, which exhibit similar characteristics to long-chain compounds but have shorter carbon chains (C > 6). Consequently, potential industrial alternatives to PFOA include perfluorobutanoic acid (PFBA, C = 4) and perfluorohexanoic acid (PFHxA, C = 6) [14,15,16,17].

Several studies conducted in the USA and European countries have confirmed the presence of PFOA in various environmental compartments (soil, air, water, including drinking water) as well as in human samples [18]. Research on the general population found an average serum PFOA level ranging from 2.3 to 3.7 ng/mL [19,20]. Studies of occupational exposure have found PFOA to be present at various concentrations, ranging from a median value of 0.0065 µg/mL in samples collected from 26 airport employees [21], to a range of 0.250–1.050 µg/mL in a group of four ski wax technicians [22], and a mean level of 4.048 µg/mL among 462 male employees who had worked before 2009 in a PFOA-producing factory (range of 0.019–91.9 ng/mL) [23]. Some reports indicate extremely high PFOA serum concentrations at occupational exposure, reaching levels as high as 12,000 ng/mL [24].

An important consideration regarding the exposure of the general population to PFCAs is the context in which accidental environmental contamination leading to increased exposure occurs. For instance, the Veneto Region in northeastern Italy was found to be contaminated with PFASs due to emissions from a manufacturing plant operating since the late 1960s. Residents were exposed to elevated levels of PFASs, primarily PFOA, through drinking water until 2013. In this case, the PFOA concentration in blood serum, measured in 5860 individuals between 2017 and 2022, was 0.049 µg/mL [25].

Studies have shown that, despite a significant reduction in industrial use, PFOA is still determined in high concentrations in body fluids like urine [26] and blood [20]. Furthermore, elevated concentrations of PFOA have been observed in tissues, suggesting accumulation in breast milk (336 ng/L; [27]) and bones (20.9 ng/g; [28]), as well as PFHxA in the liver (68.3 ng/g), brain (141 ng/g), and lungs (207 ng/g) and PFBA in the lungs (807 ng/g), brain (263 ng/g), and kidneys (263 ng/g) [28].

Human peripheral blood mononuclear cells (PBMCs) are a group of cells isolated from peripheral blood, with the largest proportion being made up of lymphocytes (T and B cells), followed by monocytes and dendritic cells. The primary role of PBMCs is to mediate an appropriate immune response in the body [29]. The optimal functionality of PBMCs is essential for maintaining an effective immune response, as impairments in this cell population may contribute to the onset of conditions such as allergies and asthma [30].

In light of the above, the goal of our study was to compare the cytotoxic and oxidative potentials of PFOA, PFBA, and PFHxA in human PBMCs (Table 1). This was accomplished through the assessment of cell viability and intracellular adenosine-5’-triphosphate (ATP) levels, as well as analyses of changes in cell morphology, generation of reactive oxygen species (ROS), highly reactive oxygen species (hROS), and reactive nitrogen species (RNS), along with lipid and protein oxidation.

## 2. Results

### 2.1. Cell Viability

**Figure 1:**
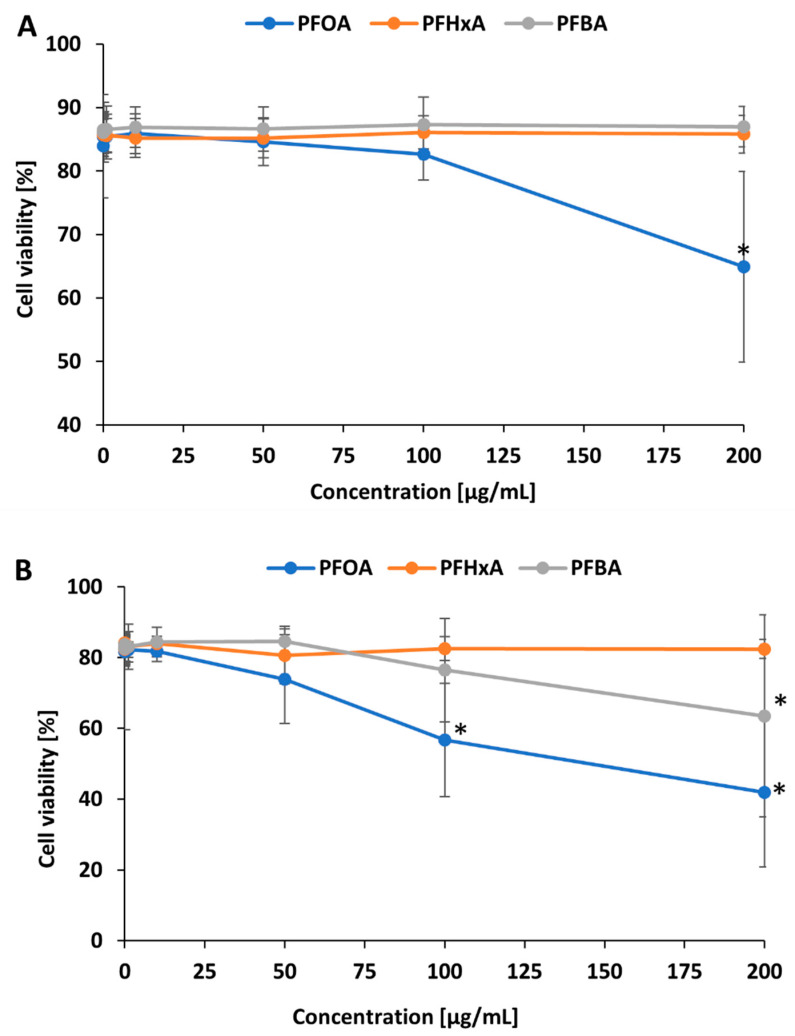
Changes in viability of human PBMCs incubated with PFOA, PFHxA, and PFBA in concentrations ranging from 0.001 to 200 µg/mL for 1 h (**A**) and 24 h (**B**). Mean ± SD was calculated from four individual experiments (four blood donors). The asterisk indicates significant difference from negative control (*p* < 0.05). Statistical analysis was performed using one-way ANOVA and Tukey’s test.

In order to determine the cytotoxicity of PFCAs, PBMCs were exposed to increasing concentrations of these compounds, and cell viability was assessed at the end of the incubation period. The percentages of viable cells in samples treated with tested compounds for 1 h and 24 h are given in Figure 1A and Figure 1B, respectively. PFOA, PFHxA, and PFBA caused various changes in the viability of PBMCs. After 1 h of incubation with PFOA at a concentration of 200 µg/mL, the viability of PBMCs significantly decreased (to 64.9 ± 15.05%) (Figure 1A). After 24 h of incubation, a statistically significant decrease in PBMC viability occurred only for PFOA at 100 µg/mL and 200 µg/mL (56.7 ± 15.99% and 41.8 ± 21.02%, respectively) and for PFBA at 200 µg/mL (63.5 ± 28.56%) (Figure 1B).

### 2.2. Cell Morphology

**Figure 2:**
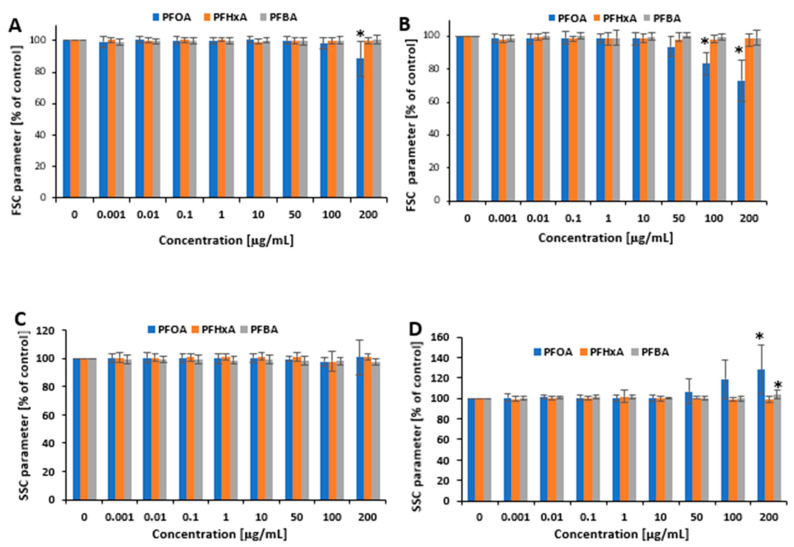
Changes in FSC for 1 h (**A**) and 24 h of incubation (**B**) and changes in SSC parameter for 1 h (**C**) and 24 h of incubation (**D**) of PBMCs with PFOA, PFHxA, and PFBA at concentrations from 0.001 to 200 µg/mL. Mean ± SD was calculated from four individual experiments (four blood donors). The asterisk indicates significant differences from negative control (*p* < 0.05). Statistical analysis was performed using one-way ANOVA and Tukey’s test.

Cell morphology was assessed by flow cytometry based on forward scatter (FSC) and side scatter (SSC) parameters. The tested compounds demonstrated different effects on FSC and SSC parameters. After 1 h of incubation, only PFOA at a concentration of 200 µg/mL caused a statistically significant reduction in FSC (88.5 ± 11.38% versus control) (Figure 2A). Similarly, after 24 h of incubation, only PFOA at 100 µg/mL and 200 µg/mL caused significant changes in FSC parameter (83.2 ± 6.79% versus control and 72.9 ± 12.43% versus control, respectively) (Figure 2B).

No statistically significant changes in SSC parameter were noted after 1 h incubation of PBMCs with PFCAs (Figure 2C). After 24 h of incubation, a statistically significant increase in SSC parameter was observed for PFOS at 100 µg/mL and 200 µg/mL (119 ± 18.98% versus control and 128.3 ± 24.16% versus control, respectively) and for PFBA at its highest tested concentration of 200 µg/mL (103.9 ± 4.73% versus control); PFHxA did not cause any statistically significant changes, even at 200 µg/mL (Figure 2D).

### 2.3. ATP Level

**Figure 3:**
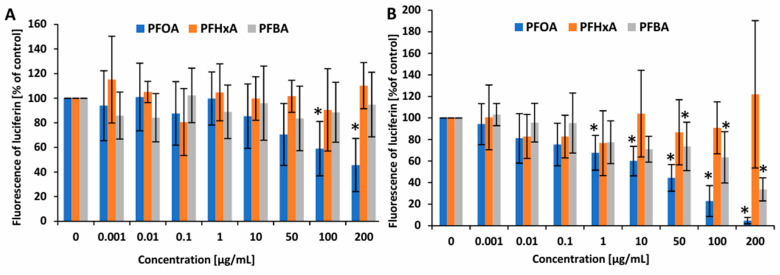
Changes in ATP level in PBMCs incubated with PFOA, PFHxA, and PFBA at concentrations ranging from 0.001 to 200 µg/mL for 1 h (**A**) and 24 h (**B**). Mean ± SD was calculated from three individual experiments (three blood donors). The asterisk indicates significant differences from negative control (*p* < 0.05). Statistical analysis was performed using one-way analysis of variance and Tukey’s test.

Intracellular ATP levels were measured to assess early cellular responses to xenobiotics, as decreased ATP may indicate impaired mitochondrial function, metabolic stress, or reduced cell viability before overt cytotoxic effects are detectable. The tested compounds altered the ATP level in PBMCs to a varying extent. After 1 h of incubation, only PFOA at 100 µg/mL (59 ± 22.09% versus control) and 200 µg/mL (45.7 ± 21.62% versus control) caused a statistically significant decrease in the ATP level, with the latter demonstrating a stronger decline (Figure 3); the differences induced by PFHxA and PFBA were statistically insignificant. 

### 2.4. Oxidative Stress Induction

**Figure 4:**
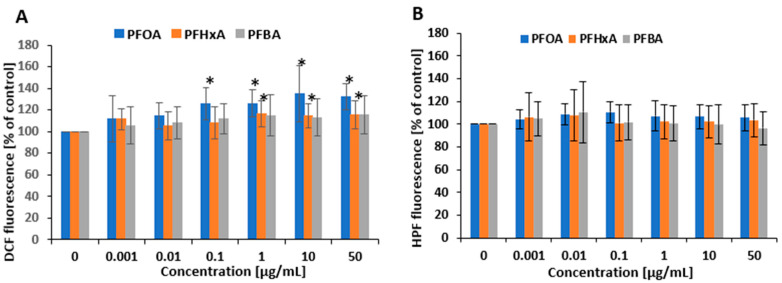
Changes in the level of ROS (**A**) and in the level of hROS (**B**) in PBMCs incubated with PFOA, PFHxA, and PFBA at concentrations of 0.001 to 50 µg/mL after 1 h of incubation. Mean ± SD was calculated from five individual experiments (five blood donors). The asterisk indicates significant difference from negative controls (*p* < 0.05). Statistical analysis was performed using one-way analysis of variance and Tukey’s test.

To determine whether short-term exposure to PFCAs induces the production of ROS and hROS (mainly hydroxyl radical) in PBMCs, thereby indicating potential oxidative stress, the cells were treated with varying concentrations of these compounds. The analyzed PFCAs affected the production of ROS in PBMCs in different manners. PFOA caused an increase in the level of ROS in human PBMCs at all concentrations from 0.1 µg/mL after 1 h of incubation. PFHxA also increased the level of ROS but in the concentration range of 1 to 200 µg/mL. For PFBA, no statistically significant changes were observed (Figure 4A). No significant changes in hROS level were noted for any PFCA (Figure 4B). The positive controls for ROS and hROS reached values of 206.1 ± 47.9% and 119.8 ± 14.67%, respectively.

### 2.5. Nitrosative Stress Induction

**Figure 5:**
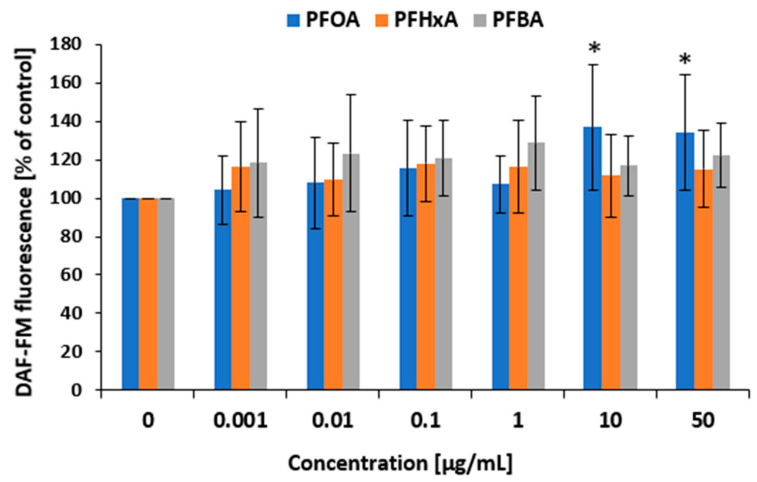
Changes in the level of RNS in PBMCs incubated with PFOA, PFHxA, and PFBA at concentrations of 0.001 to 50 µg/mL for 1 h. Mean ± SD was calculated from four individual experiments (four blood donors). The asterisks indicate significant difference from negative controls (*p* < 0.05). Statistical analysis was performed using one-way analysis of variance and Tukey’s test.

To confirm whether RNS production may occur after PBMC treatment with selected PFCAs, a test with diaminofluorescein-FM diacetate (DAF-FM DA) was conducted. The positive control (SNP) reached values of 125.2 ± 30.98%. Changes in the levels of RNS were noted after 1 h of treatments with PFOA, PFHxA, and PFBA (Figure 5). It was observed that only PFOA increased the level of RNS in a significant manner at its highest tested concentrations of 10 µg/mL and 50 µg/mL (136.9 ± 32.64% versus control and 134.1 ± 30.13% versus control).

### 2.6. Protein Damage

**Figure 6:**
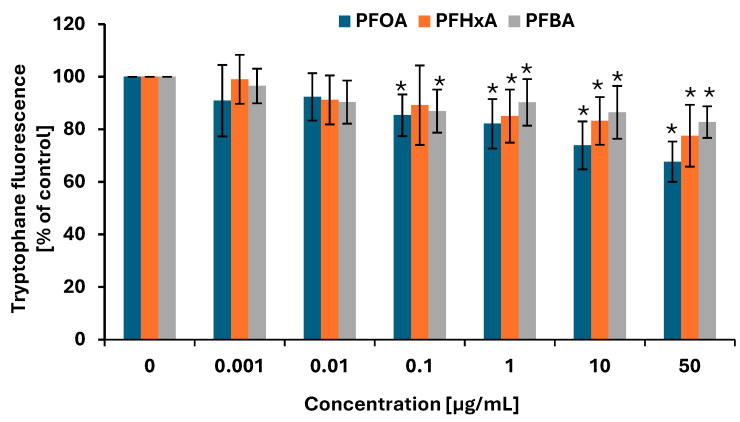
Changes in protein damage (mainly oxidation of tryptophan) in PBMCs incubated with PFOA, PFHxA, and PFBA in concentrations ranging from 0.001 to 50 µg/mL for 1 h. Mean ± SD was calculated from four individual experiments (four blood donors). The asterisks indicate significant differences from negative control (*p* < 0.05). Statistical analysis was performed using one-way analysis of variance and Tukey’s test.

Protein oxidation is one of the key indicators in oxidative stress analysis, as oxidative modifications can impair protein structure and function, thereby reflecting the extent of cellular oxidative damage. The analyses were performed using the fluorescent properties of tryptophan. A significant decrease in tryptophan fluorescence was noted in PBMCs incubated with PFOA, PFHxA, and PFBA after 1 h of treatment (Figure 6), indicating that the tested compounds induced protein damage in the studied cells. Significant increases in protein damage were noted for the both long- and short-chain PFCAs at concentrations ranging from 0.1 to 50 µg/mL (excluding PFHxA at 0.1 µg/mL). PFOA and PFBA at 0.1 µg/mL reduced tryptophan fluorescence up to 85.4 ± 7.93% compared with controls and 86.9 ± 8.21% compared with controls, respectively.

### 2.7. Lipid Peroxidation

**Figure 7:**
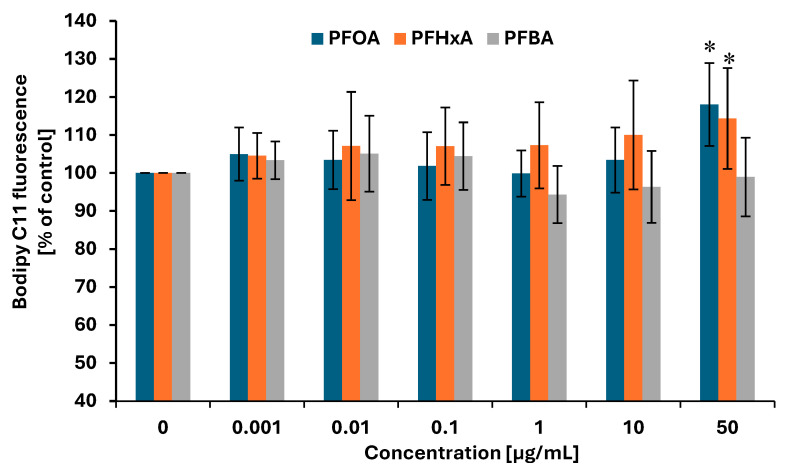
Changes in lipid peroxidation in PBMCs incubated with PFOA, PFHxA, and PFBA at concentrations of 0.001 to 50 µg/mL for 1 h. Mean ± SD was calculated from seven individual experiments (seven blood donors). The asterisks indicate significant differences from negative control (*p* < 0.05). Statistical analysis was performed using one-way analysis of variance and Tukey’s test.

Lipid peroxidation plays a critical role in oxidative stress by compromising membrane lipid integrity, leading to altered membrane fluidity, increased permeability, and subsequent impairment of cellular homeostasis and signaling pathways. Lipid peroxidation was assessed in PBMCs based on the fluorescence method with boron dipyrromethene difluoride (Bodipy 581/591 C11) conjugated with fluorescein. It was observed that both long (PFOA)- and short-chain PFCAs (PFHxA) induced significant increase in Bodipy fluorescence at the highest concentration of 50 µg/mL (118.0 ± 10.91% versus control and 114.4 ± 13.26% versus control, respectively) (Figure 7). Luperox used as a positive control induced strong lipid peroxidation (801.1 ± 858% versus control).

## 3. Discussion

Most research on PFASs has focused on those with longer chains, i.e., with carbon chains consisting of 8 to 10 atoms. Consequently, the effects of short-chain PFAS compounds such as PFCAs, particularly concerning their impact on human health and the immune system, remain relatively limited, despite increasing evidence of the harmful effects on living organisms.

This study compared the potentials of PFOA and its two industrial substitutes, viz., PFHxA and PFBA, to induce cytotoxic and oxidative stress on PBMCs. PBMCs play a critical role in the human body by maintaining homeostasis and mediating in immune responses against pathogens and cancer cells [31]. Our results indicate that the tested compounds affect PBMC viability to differing degrees (Figure 1A,B). PFOA was found to induce the most pronounced reduction in cell viability, noted at 200 µg/mL after 1 h of incubation and at 100 µg/mL and 200 µg/mL after 24 h of incubation. Interestingly, PFBA reduced cell viability at its highest tested concentration after 24 h of incubation, whereas no significant alterations were noted for PFHxA. The findings reported by Amstutz et al. [32] in THP-1 cells showed that PFOA, PFHxA, and PFBA decreased cell viability after 3 h of incubation at a comparable concentration, i.e., 100 µg/mL. Notably, consistent with our findings, PFBA exerted a stronger effect on viability than PFHxA. PFBA may exhibit higher toxicity than PFASs with longer chains (e.g., PFHxA or PFOA) for various reasons. For example, PFBA is a short-chain compound with a lower molecular weight; as such, at a given concentration of exposure (e.g., 50 µg/mL), it will interact with a larger number of molecules than a longer-chain compound and engage in more intense interactions with membranes or enzymes. Additionally, the stronger hydrophilicity and smaller particle size of PFBA facilitate its transport across cell membranes [33]. PFBA can thus penetrate more rapidly into cells, disrupting their metabolism and inducing oxidative stress. PFBA also has a higher pKa value than PFOA and PFHxA, and it has been suggested that a small number of its molecules exist in an electronegative form; this allows it to potentially mimic a small neutral fatty acid, which may disrupt the lipid metabolism of the cell [34]. Furthermore, it has been shown that, compared with PFOA or PFHxA, PFBA can preferentially modulate various activation pathways such as the farnesoid X receptor/retinoid X receptor signaling pathway (FXR/RXR)—a transcriptional system playing a crucial role in lipid, cholesterol, and bile acid homeostasis [35].

The tested compounds exhibited distinct effects on FSC and SSC parameters analyzed by flow cytometry, which are indicative of changes in cell size and granularity, respectively. Among the tested compounds, PFOA was the only one to induce alterations in both parameters. After 1 h incubation, PFOA at 200 µg/mL decreased FSC; however, at 100 µg/mL and 200 µg/mL, it more strongly decreased FSC and increased SSC after 24 h of incubation. In contrast, for the other short-chain substitutes, only PFBA demonstrated any changes: SSC was found to increase in cells incubated at the highest concentration for 24 h (Figure 2A–D). Changes in FSC and a reduction in PBMC viability were noted at the same significant concentrations, which may indicate that PFOA and PFBA caused loss in cell membrane integrity, which influenced necrotic cell death. In turn, the observed increase in SSC may indicate an increased number of damaged organelles in dying cells [36,37,38].

Mitochondrial dysfunction results in a reduction in ATP production within cells, which in turn can lead to cellular dysfunction and ultimately cell death [39]. Alterations in ATP levels may occur as part of aging-related processes but can also be induced by exposure to certain toxic substances [40]. The analysis of cellular ATP levels can serve as an accurate indicator of cell viability, as it reflects overall metabolic activity and mitochondrial function. It is considered to be a more sensitive parameter than classical cell viability analysis, as a decrease in ATP level can occur at an early stage of cell damage, even before cell membrane integrity is lost or cell proliferation is inhibited [41]. These observations were confirmed in our analyses, where statistically significant changes were noted at concentrations lower than those observed in cell viability analyses. In the present study, a significant decrease in ATP levels was observed after 1 h of incubation exclusively for PFOA at 100 µg/mL and 200 µg/mL (Figure 3A,B). Considering that ATP serves as a fundamental energy source for essential metabolic processes and cellular function, it can be postulated that the observed reduction in ATP levels induced by PFOA contributed to the previously described necrotic death of PBMCs. Similar findings were reported by Mashayekhi et al. [42], who demonstrated that exposure of rat liver and brain mitochondria to PFOA at concentrations ranging from 0.5 to 1.5 mM resulted in a concentration-dependent decrease in intracellular ATP levels. Furthermore, the reduction in ATP content was found to be more pronounced in liver mitochondria than in brain mitochondria.

ROS and RNS are reactive forms generated under physiological conditions during oxidative metabolism in cells; they are involved in cell signaling, the regulation of cell survival and death, or the production of factors associated with inflammation [43,44]. The pro- and antioxidant balance is tightly controlled in the cell to avoid increased oxidative stress. In the present study, the greatest increase in ROS formation was observed for long-chain PFOA after 1 h of incubation, from a concentration of 0.1 ug/mL. A significant increase in ROS generation was also noted in PBMCs incubated with PFHxA from a concentration of 1 µg/mL; however, no significant changes were observed in those incubated with PFBA (Figure 4A). Available literature data have suggested that PFHxA can induce ROS production in certain cell types, such as HepaRG and monocytes [32,45], indicating that this the compound can induce oxidative stress. Nevertheless, it is difficult to conclusively link its pro-oxidative action to its toxicity to the mitochondrion, as studies in this area are relatively few and inconsistent.

However, the effect of PFHxA on other oxidative parameters, such as lipid peroxidation or protein oxidation, is worth noting. It has been shown that exposure can disrupt lipid homeostasis in hepatic cells such as HepaRG, affecting lipid composition and fat metabolism [32]. In addition, PFHxA may indirectly generate ROS by activating PPARα receptor-related pathways, which regulate lipid metabolism and redox reactions. Fujiwara et al. [46] report that PFOA at the highest analyzed concentration (200 µM, 500 µM, and 600 µM) caused a significant increase in ROS level in mouse ameloblast-lineage cells (ALCs). Some authors suggest that observed PFOA-mediated cell necrosis is induced by ROS-MAPK/ERK signaling. A study by Suh et al. [47] showed that PFOA significantly increased ROS levels, mitochondrial superoxide, nitric oxide, and pro-inflammatory cytokine production in pancreatic β-cell-derived RIN-m5F cells. Additionally, PFOA triggered a reduction in ATP level, which was also noted in the present study. The effect of increased ROS production induced by selected perfluoroalkyl compounds has also been demonstrated in numerous in vitro and in vivo studies [32,45,48].

Interestingly, some studies have indicated that PFBA enhances oxidative stress to a greater extent than PFOA. A study conducted on human neuronal cells (SH-SY5Y line) demonstrated that a 24 h incubation with PFOA and PFBA at 1 µg/mL caused increased ROS production. However, PFBA induced more pronounced changes in ROS levels and caused a greater reduction in antioxidant enzyme activity compared with PFOA [49]. The observed differences can be attributed to the use of different cellular models in the experiments (primary, physiologically relevant cells vs. cancer cell lines), which exhibit distinct metabolism and resistance to xenobiotics.

The hydroxyl radical is one of the most highly reactive ROS, and its elevated levels within the cell can lead to cell death through the damage of proteins, lipids, and DNA [50]. In the present study, none of the analyzed compounds were found to induce an increase in hydroxyl radical production in PBMCs following 1 h incubation (Figure 4B). Our findings also indicate that only PFOA was capable of elevating RNS (nitric oxide) level with a significant increase noted from a concentration of 10 µg/mL (Figure 5). The ability of PFOA to induce nitrosative stress has been previously observed in both in vitro [37,51] and in vivo studies [52]. However, there currently appears to be no clear evidence indicating that PFBA or PFHxA directly affect the formation of RNS, inducing nitrosative stress. While one study reported that PFBA influences oxidative stress-induced metabolic and transcriptional reprogramming, including nitrogen metabolism, it was conducted on wheat seedlings, i.e., plant cells [53].

Uncontrolled oxidative stress can lead to amino acid modifications in proteins, oxidation of thiol groups, and protein aggregation, thus impairing specific enzymatic functions and disrupting key signaling pathways [54]. Our present findings indicate an increase in protein oxidation for all tested compounds, with significant effects starting from a concentration as low as 0.1 µg/mL for PFOA and PFBA and from 1 µg/mL for PFHxA (Figure 6). Obiako et al. [49] demonstrated that PFOA and PFBA reduce the activity of key antioxidant enzymes, including catalase, glutathione reductase, and lactate dehydrogenase, in SH-SY5Y cells. Furthermore, Zhang et al. [16], using molecular docking analysis in studies on embryonic and adult zebrafish, showed that PFOA and its short-chain substitutes stably bind to proteins, altering their structure and function.

Increased oxidative stress can also lead to lipid peroxidation and subsequent lipid damage [55], resulting in the loss of cellular membrane integrity and alterations in its composition, structure, and dynamics. Lipid peroxides formed during lipid peroxidation have been shown to promote ROS formation and degrade molecules responsible for protein and DNA cross-linking [56]. Our present findings also indicate significant increases in lipid peroxidation in PBMCs exposed to PFOA and PFHxA, although only at relatively high concentrations, i.e., 50 µg/mL (Figure 7). Consistently, Endirlik et al. [57] reported elevated levels of malondialdehyde (MDA), a marker of lipid peroxidation, in the brains of Balb/c mice following PFOA administration at a significant dose of 30 mg/kg. Similarly, Alamo et al. [58] demonstrated a dose-dependent increase in lipid peroxidation in human sperm cells exposed to PFOA at 0.01 mM, 0.1 mM, and 1 mM.

The correlation between PFCA toxicity and chain length remains a subject of ongoing debate; however, the majority of in vitro and in vivo studies have suggested that long-chain compounds exhibit greater toxicity than their short-chain alternatives [35,59,60,61]. Our study also demonstrated that PFOA exerted the strongest effect on the analyzed parameters. The toxicity of PFOA may be attributed to several factors. Primarily, its longer carbon chain (C = 8) increases hydrophobicity, facilitating its retention within the cell membrane and interaction with its components. Our findings indicate that only PFOA and PFHxA, i.e., the compounds with the longest carbon chains, induced lipid peroxidation of PBMCs, supporting the hypothesis that higher membrane retention contributes to their effects. It is, however, important to emphasize that PFBA (C = 4) and PFHxA (C = 6) caused less pronounced changes in all tested parameters in comparison with PFOA (C = 8) in the tested cells; this challenges the assumption that PFCA toxicity is strictly correlated with carbon chain length.

Interestingly, Sobolewski et al. [62] reported that PFOA, even at nanomolar concentrations, induced modifications in model vesicles composed of a lipid bilayer, promoting the secondary accumulation of solutes. This finding suggests that PFCA molecules retained within the cell membrane may facilitate the preferential accumulation of additional molecules of the same compound, potentially exacerbating their biological effects.

Similarly, a study of PFAS toxicity by Amstutz et al. [32] found PFOA to demonstrate greater ROS-generating potential in monocytes than PFHxA and PFBA, indicating that the longest carbon chain compound had the highest toxicity. Interestingly, some studies suggest that PFOA may be less toxic than their shorter chain analogues. An in vivo study on embryonic and adult zebrafish by Zhang et al. [16] showed that PFHxA exhibited greater toxicity than PFOA, suggesting that the significant toxicity of PFBA may be attributed to its shorter carbon chain; this would enhance cellular permeability, leading to higher intracellular concentrations and, consequently, more pronounced biological effects.

It is evident that the toxicity of PFBA and PFHxA may vary depending on the type of cell or tested species, exposure conditions, and experimental parameters. Therefore, further research is essential to fully elucidate the mechanisms underlying the observed differences in the toxicity of these compounds.

Our data indicate that the lowest concentration of PFCAs to yield statistically significant effects is 0.1 µg/mL. While this concentration may be relevant to occupational or environmental exposure scenarios, it is unlikely to occur in the general population except in cases of extremely high accidental contamination of residential areas. PBMCs are known to exhibit donor-dependent variability in response to xenobiotics, influenced by factors such as age, sex, and genetic predisposition, which may limit the generalizability of the findings. Taking into account that these variables were not analyzed in the present study and the number of blood donors was relatively limited (ranging from three to seven), appropriate measures were implemented to ensure the quality and consistency of the study. All donors fulfilled the official eligibility criteria established by the blood donation center, comprising healthy, non-smoking individuals aged 18 to 55 years, free from any acute infection at the time of donation. Experimental procedures were meticulously standardized, and only the most reproducible datasets were included in the final analysis. Although this approach mitigates inter-donor variability to some degree, it does not completely eliminate it. Future studies encompassing a larger number of blood donors are necessary to validate and extend the present findings. Nonetheless, the current results provide valuable preliminary insights into the potential immunotoxic effects of PFCAs on human PBMCs and lay the groundwork for further investigation.

## 4. Materials and Methods

### 4.1. Chemicals

PFOA, (purity ≥ 95%), PFHxA, (purity ≥ 98%), PFBA (purity ≥ 98%), propidium iodide, calcein-AM, fetal bovine serum (FBS), penicillin–streptomycin, calcein-AM (95%), propidium iodide (PI, 95%), and 3′-(p-hydroxyphenyl)-fluorescein (HPF) were purchased from Sigma-Aldrich (St. Louis, MO, USA). RPMI 1640 with L-glutamine, Bodipy, 4-amino-5-methylamino-2′,7′-difluororescein (DAF-FM), 2′,7′-dichlorodihydrofluorescein diacetate (H_2_DCFDA), and Luperox were purchased from Thermo Fisher (Nijmegen, The Netherlands). The ATP assay kit was bought from Molecular Probes (Eugene, OR, USA). Lymphocyte separation medium (LSM, 1.077 g/cm^3^) was bought from Cytogen (Greven, Germany). Other chemicals were obtained from Carl Roth (Karlsruhe, Germany) and POCH (Gliwice, Poland).

### 4.2. Methods

#### 4.2.1. Cell Isolation and Treatment

PBMCs were prepared from a leukocyte–platelet buffy coat obtained from the Regional Centre for Blood Donation and Treatment (RCBDT) in Lodz, Poland, accredited by the Ministry of Health (Accreditation No. BA/2/2004). 

Venous blood was collected in the RCBDT from healthy, non-smoking individuals aged 18–55. PBMCs were separated from the buffy coat by the density gradient method using a lymphocyte separation medium (LymphoSep, 1.077 g/cm^3^). These samples were centrifuged without a brake (3000 rpm, 10 min, 20 °C). The collected PBMCs were then mixed with Na_2_PBS, layered over LSM in new tubes, and centrifuged again (3000 rpm, 30 min, 20 °C) without a brake. PBMCs were carefully transferred to fresh tubes and suspended in erythrocyte lysis buffer (150 mM NH_4_Cl, 10 mM NaHCO_3_, 1 mM EDTA, pH 7.4), mixed thoroughly, and incubated for 5 min at 20 °C. In the next step, to neutralize the lysis buffer, 6.5 mL PBS per probe was added immediately and mixed, and the suspension was centrifuged at 600 rpm for 15 min at 20 °C. Then, the supernatant was discarded, and the wash was repeated twice with RPMI 1640 supplemented with L-glutamine, 10% fetal bovine serum (FBS), and 0.5% penicillin–streptomycin (600 rpm, 15 min, 20 °C). Finally, the PBMCs were resuspended in RPMI 1640 medium with the specified supplements. The final PBMC density used in the assays was 1 × 10^6^ cells/mL.

The cells were treated with the tested PFCAs for 1 h or 24 h, depending on the variant. In the experiments analyzing oxidative stress parameters, it was decided to use a 1 h incubation period to capture the direct effect of the xenobiotics without interference from secondary processes such as activation of stress response pathways or initiation of cell death, as these could interfere with the interpretation of early cellular responses. The applied concentrations ranged from 0.001 to 200 µg/mL for the viability and ATP test and from 0.001 to 50 µg/mL for the others. The samples were incubated in complete darkness, in a 5% CO_2_ atmosphere at 37 °C. Concentrations of 0.001 µg/mL and 0.01 µg/mL were chosen as the lowest concentrations: these are believed to correspond to levels found during environmental exposure to these compounds [20]. PFCA concentrations of 0.1 µg/mL and above are considered to correspond to occupational exposure levels [24].

The test substances were dissolved in DMSO. This was present at a concentration of 0.4% in the negative control samples and in the samples treated with PFOA, PFHxA, or PFBA. The concentration of DMSO used in the experiments was non-toxic to PBMCs, as confirmed by all assessed parameters.

#### 4.2.2. Cell Viability

Cell viability was assessed using two fluorescent markers: calcein-AM (excitation/emission maxima: 494/517 nm) and propidium iodide (PI, excitation/emission maxima: 535/615 nm). Calcein-AM is hydrolyzed to calcein, which carries a negative charge and enters live cells, staining them green. Propidium iodide enters cells with compromised membrane integrity (necrotic and dead cells) and binds to DNA, staining them red [63].

PBMCs were incubated with PFOA, PFHxA, or PFBA at concentrations ranging from 0.001 to 200 µg/mL for 1 h and 24 h. After incubation, the cells were centrifuged at 1500 rpm for 5 min at 4 °C, and the supernatant was removed. Then, the cells were resuspended in RPMI 1640, and calcein-AM (50 nM) and PI (1 µM) were added to the samples. After incubation (15 min at 37 °C in the dark), fluorescence was measured using a flow cytometer (LSR II, BD Biosciences, San Jose, CA, USA). The major viability analysis was preceded by compensation using untreated, unstained cells (for gating the PBMC population); cells stained only with calcein-AM (for gating live cells); and cells stained only with propidium iodide after ethanol treatment (40%) (for gating dead cells). The FCM gate for PBMCs was set for data acquisition, and the measurement was performed for 10,000 cells.

#### 4.2.3. Cell Morphology

Cell morphology was analyzed using flow cytometry. The intensity of FSC correlates with cell size, while scattering at a 90° angle, measured as SSC, increases proportionally to the content of intracellular structures (such as granules) that reflect light. The analysis of these parameters allows for the assessment of changes in the size and granularity of the examined cells. As a cell passes through the focused laser beam of the cytometer, it scatters light. The analysis of this scattering provides information on the size, shape, and structure of the cells.

PBMCs were incubated with PFOA, PFHxA, or PFBA at concentrations ranging from 0.001 to 200 µg/mL for 1 h or 24 h. Following this, the cells were centrifuged at 1500 rpm for 5 min at 4 °C, and the supernatant was removed. The cell suspension was mixed with 500 μL of fresh RPMI 1640 media solution for flow cytometric measurement. The FCM gate on PBMCs was set for data acquisition, and the measurement was performed using an LSR II flow cytometer (LSR II, Becton Dickinson, Franklin Lakes, NJ, USA). The obtained data were presented as a diagram plotting cell number against light scatter and analyzed using WinMDI 2.8 software.

#### 4.2.4. ATP Level

The intracellular level of ATP was determined by oxidative decarboxylation of D-luciferin by luciferase in the presence of ATP and magnesium ions with simultaneous bioluminescence.

The level of the intracellular ATP level was tested using the ATP assay kit (Molecular Probes) according to the manufacturer’s instructions.

After incubation of PBMCs for 1 or 24 h with the appropriate compounds at concentrations of 0.001–200 µg/mL, the cells were centrifuged at 15,000 rpm for 10 min at 4 °C, and the supernatant was discarded. Cellular ATP was then extracted by adding 1 mL of boiling deionized water to the cells and shaking the samples [64]. The samples were vortexed and centrifuged (15,000 rpm for 15 min at 4 °C); following this, 30 µL of supernatant from each sample was transferred to a 96-well plate, and 100 µL of luciferin/luciferase mixture was added. The samples were incubated for 20 min at room temperature in complete darkness. Bioluminescence was measured at 560 nm for 0.1 min (Fluoroskan Ascent FL, Labsystems, Vantaa, Finland).

#### 4.2.5. ROS Measurement

ROS content was determined using 2,7-dichlorodihydrofluorescein diacetate (H_2_DCFDA). H_2_DCFDA, a non-fluorescent compound, is hydrolyzed by membrane esterases to form 2′,7′-dichlorodihydrofluorescein (H_2_DCF), which is also non-fluorescent. Upon entering the cell membrane, H_2_DCF is oxidized to 2′,7′-dichlorofluorescein (DCF), whose fluorescence is analyzed at excitation and emission maxima of 488 nm and 530 nm, respectively.

The PBMCs were incubated for 1 h with PFOA, PFHxA, or PFBA at concentrations ranging from 0.001 to 50 µg/mL. Then, the cells were centrifuged at 1500 rpm for 5 min at 4 °C, the supernatant was removed, and PBMCs were resuspended in PBS. Finally, H_2_DCFDA (5 µM) was added to the samples, which were incubated for 20 min at 37 °C in total darkness. To induce ROS production, the cells were treated with 200 µM hydrogen peroxide for 15 min (positive control). The measurement was performed using an LSR II flow cytometer (Becton Dickinson). During the analysis, an FCM gate was set up for data acquisition, and the data were recorded for a total of 10,000 events per sample.

#### 4.2.6. hROS Measurement

The intracellular hROS level was determined using the fluorescent dye 3′-(p-hydroxyphenyl)-fluorescein (HPF). HPF is preferentially oxidized by intracellular hydroxyl radicals into a highly fluorescent product (excitation/emission maxima 490/515 nm) [65,66].

PBMCs were incubated for 1 h with PFOA, PFHxA, or PFBA at concentrations ranging from 0.001 to 50 µg/mL. The cells were centrifuged at 1500 rpm for 5 min at 4 °C. The supernatant was removed, and PBMCs were resuspended in PBS. Finally, HPF was added (4 µM) to the samples, which were incubated for 20 min at 37 °C in the dark. The cells treated with a mixture of iron(II) perchlorate (0.1 mM) and hydrogen peroxide (1 mM) were used as a positive control. Fluorescence was measured using an LSR II flow cytometer (Becton Dickinson) at maximum excitation/emission wavelengths of 490 nm and 515 nm, respectively. During the analysis, an FCM gate was set up for data acquisition, and the data were recorded for a total of 10,000 events per sample.

#### 4.2.7. RNS Measurement

Diaminofluorescein-FM diacetate (DAF-FM DA) is a fluorescent dye used to detect intracellular nitric oxide (NO) level. DAF-FM DA is a cell-permeable deacetylated form of DAF-FM, which is hydrolyzed by intracellular esterases to form cell-impermeable DAF-FM. In the presence of oxygen, DAF-FM is oxidized to a triazole-fluorescein analogue (DAF-FM T), which exhibits approximately 160-fold greater fluorescence quantum yield.

PBMCs were incubated for 1 h with PFOA, PFHxA, or PFBA at concentrations ranging from 0.001 to 50 µg/mL. After incubation, the cells were centrifuged (1500 rpm, 5 min, 4 °C). The supernatant was removed, and PBS was added to each sample. Following this, DAF-FM DA was added to each sample and incubated for 20 min at 37 °C in total darkness. Sodium nitroprusside/SNP, 300 µM, was used as a positive control. After incubation, the samples were measured using an LSR II flow cytometer (Becton Dickinson) at maximum excitation/emission wavelengths of 500 nm and 515 nm, respectively. During the analysis, an FCM gate was set for data acquisition, and the data were recorded for a total of 10,000 events per sample.

#### 4.2.8. Protein Damage

The fluorescence properties of proteins are associated with the presence of aromatic amino acids, particularly tryptophan, in their structure. A reduction in fluorescence at 335 nm indicates oxidative damage to tryptophan, thereby reflecting protein damage in PBMCs [67]. Protein oxidation analysis was performed as described previously [68].

#### 4.2.9. Lipid Peroxidation

A Bodipy 581/591 C11 (conjugated with fluorescein) probe was used to evaluate lipid peroxidation levels. This technique is based on a fluorescence shift induced by dye oxidation. In its unoxidized form, the dye emits red fluorescence at approximately 590 nm. However, when oxidized by superoxide radicals, which are by-products of lipid peroxidation, the dye undergoes a fluorescence shift, emitting green fluorescence at around 510 nm. As the extent of this fluorescence change is directly correlated with the degree of lipid peroxidation, it can be used to assess the degree of lipid oxidation in the samples. Lipid peroxidation levels were evaluated according to Xiao and Quing [69].

The PBMCs were incubated for 1 h with PFOA, PFHxA, or PFBA at concentrations ranging from 0.001 to 50 µg/mL. Following this, the cells were removed, and Bodipy was added to the cells (1.25 µM) and incubated for 1 h at 37 °C in total darkness. Then, the samples were centrifuged (1500 rpm, 5 min, 4 °C), and the supernatant was removed. PBS was added to the samples, gently mixed, and the fluorescence (FITC, excitation/emission maxima: 488/510 nm) was measured using an LSR II flow cytometer (Becton Dickinson). Cells treated with Luperox (tert-butyl hydroperoxide, 50 µM) were used as a positive control. During the analysis, an FCM gate was set for data acquisition, and the data were recorded for a total of 10,000 events per sample.

#### 4.2.10. Statistical Analysis

Data are expressed as mean values with standard deviation. The relationships were tested based on comparison of means using one-way analysis of variance (ANOVA). The differences between the tested samples were sampled using Tukey’s multiple comparison post hoc test. Statistical significance was *p* < 0.05. Data normality was evaluated by the Shapiro–Wilk test. The studies were performed on blood from three to seven donors. For each individual experiment (one blood donor), the experimental point was the mean value from three repetitions. The analysis was performed using STATISTICA 13 software (StatSoft, Inc., Tulsa, OK, USA).

## 5. Conclusions

PFCAs, such as PFOA, PFHxA, and PFBA, cause adverse effects in human PBMCs. The tested compounds were found to reduce PBMC viability, alter cell morphology, and deplete ATP level. It may be concluded that tested compounds are more likely to induce oxidative than nitrosative stress in PBMCs, which results in damage to lipids and, to a greater extent protein, in PBMCs. Although PFOA was found to have the strongest effect on the tested parameters, the mechanism of action of long- and short-chain PFCAs is not strictly dependent on carbon chain length. The changes were observed at concentrations that may arise from environmental exposure, but some (ROS formation, protein damage) may potentially occur in the human body as a result of occupational exposure.

The present study provides novel insights into the biological effects and mechanisms affected by selected PFCAs in PBMCs; however, it was only intended as basic research to explore the potential effects of PFOA, PFHxA, and PFBA on selected immune system cells. As the design does not follow a cohort study format, factors such as donor age, sex, or genetic background may have influenced the observed responses and could contribute to variability in the results. As the current evidence is not yet sufficient for definitive conclusions, further research is required to obtain a comprehensive understanding of the effects of PFASs on PBMCs, particularly those based on more in-depth epidemiological and mechanistic research.

## Figures and Tables

**Table 1:** List of tested PFCAs.

PFOA	PFHxA	PFBA
CAS: 335-67-1	CAS: 307-24-4	CAS: 375-22-4
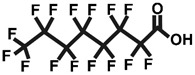	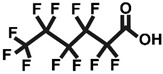	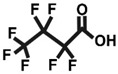

## Data Availability

Data will be made available on request.
